# Trajectories and predictors of adolescent purpose development in self‐driven learning

**DOI:** 10.1111/cdev.14201

**Published:** 2024-11-21

**Authors:** Kaylin Ratner, Hou Xie, Gaoxia Zhu, Melody Estevez, Anthony L. Burrow

**Affiliations:** ^1^ University of Illinois Urbana‐Champaign Champaign Illinois USA; ^2^ National Institute of Education at Nanyang Technological University Singapore; ^3^ GripTape New York City New York USA; ^4^ Cornell University Ithaca New York USA

## Abstract

Purpose offers several important benefits to youth. Thus, it is necessary to understand *how* a sense of purpose develops in supportive contexts and *what* psychological resources can help. From 2021 to 2022, this study investigated purpose change among 321 youth (*M*_age_ = 16.4 years; 71% female; 25.9% Black, 33.3% Asian, 15.6% Hispanic/Latinx, 13.4% White, 9.7% multiracial) participating in *GripTape*, a ~10‐week self‐driven learning program. Many youth started with high initial purpose that increased throughout enrollment (*Strengthening*), whereas others began with slightly lower purpose that remained stable (*Maintaining*). For each unit increase in baseline agency, youth were 1.6x more likely to be classified as *Strengthening*. As such, agency may be a resource that helps youth capitalize on certain types of environments.

**Abbreviations**
AIC: Akaike information criterion
BIC: Bayesian information criterion
CFI: comparative fit index
LCGA: latent class growth analysis
LMR: LoMendel‐Rubin
LRT: likelihood ratio test
RMSEA: root mean square error of approximation
SABIC: sample‐size‐adjusted Bayesian information criterion
SCS: smallest class size
SRMR: standardized root mean square residual
TLI: Tucker–Lewis index
USD: U.S. dollars
VIF: variance inflation factor
VLMR: Vuong‐LoMendell‐Rubin

## Trajectories and predictors of adolescent purpose development in self‐driven learning

Defined as an overarching life aim (Ryff, 1989), a sense of purpose in life has been nominated as a robust asset for adolescent development (Hill & Burrow, 2021; Scales et al., 2000). Purposeful youth are not only more intentional in tasks like identity exploration (Bronk, 2011; Hill & Burrow, 2012), but they also tend to be happier (Burrow & Hill, 2011). However, research on the benefits of purpose has outpaced the study of environments that support its development. Thus, we know little about (a) how purpose changes in potentially favorable environments and (b) what resources support purpose when navigating such environments. If a sense of purpose holds as much promise for youth development as prior literature suggests, needed are studies that help us understand how purpose unfolds in supportive settings and what resources help adolescents seize developmental opportunities when they are given.

This study begins to address both gaps. First, drawing on 10 weeks of data, we investigated whether multiple trajectories of purpose development exist within a unique out‐of‐school time program that supports adolescents' autonomous learning and exploration of self‐identified passions (*GripTape*; https://www.griptape.org). By enabling youth to pursue the topics they deem personally important, such an environment may be useful for promoting purpose. Then, using data collected from youth at baseline, we identified predictors of purpose trajectory within this program. In addition to offering greater insight into adolescent purpose development, this work will identify strengths that help youth capitalize on what certain contexts can offer.

## Defining adolescent purpose

Conceptual issues plague the purpose literature. Therefore, it is important to understand points of overlap and disagreement in this area of scholarship before exploring how purpose might develop in supportive environments.

Early definitions of purpose described the construct as a superordinate life aim, imbuing life with a sense of self‐direction and intentionality, and giving rise to the sense that life has meaning (Ryff, 1989). Later conceptualizations of purpose (McKnight & Kashdan, 2009) extended this definition further into the literature on goals and motivation. Here, it was suggested that purpose is fueled by personal agency and serves to direct, select, and distribute resources to personally significant pursuits. Goals are linked with a sense of purpose (e.g., Lewis, 2020; Ratner et al., 2024), but they are distinct concepts: whereas goals are proximal, concrete objectives meant to be achieved (e.g., “to become a doctor”), purpose is thought to be a distal and abstract aim with no fixed endpoint (e.g., “to help people”). Thus, a sense of purpose can organize several consonant goals around a distant objective. When purpose was introduced to the literature on youth development, however, it started being defined by three criteria (Damon et al., 2003). Like the above, purpose was thought to be (1) a significant long‐term intention that (2) a person was actively engaged in pursuing, evidenced by the commitment of time, resources, and energy to the given intention. But, unlike previous definitions, Damon an colleagues (2003) suggested that (3) purpose must also include “an external component, the desire to make a difference in the world, [and] to contribute to matters larger than the self” (p. 121). While the former definitions of purpose can be found readily throughout adult‐based research, this “beyond‐the‐self” version of purpose has been the most popular conceptualization of purpose in youth‐based research to date.

Despite these nuances in how purpose is defined throughout the literature, definitions typically agree that purpose connects to one's identity, embodying one's core values and beliefs (e.g., Kashdan & McKnight, 2009; McKnight & Kashdan, 2009). Furthermore, there seems to be agreement that purpose involves a future orientation, volition, long‐term intentionality, behavioral engagement, and goal relevance. Qualitative evidence has further suggested that adolescents and young adults generally agree with this conceptualization of purpose, describing it with themes of directionality, motivation, intention, and personal significance when prompted (Hill et al., 2010; Ratner et al., 2021). An external component is often mentioned in youth conceptions of purpose, but it seems to occur less frequently than the central willfulness themes named above.

Still, the fracture in the purpose literature has given rise to what scholars have described as sense versus content approaches (Burrow et al., 2021). Under the sense approach, subjective feelings of purposefulness are privileged and typically measured with self‐report tools. This approach tends to be agnostic to what the individual is pursuing, so long as it is energizing and provides a sense of life direction. The content approach, in contrast, is much more focused on the themes that emerge within one's purpose narrative. Purpose is typically assessed through qualitative interviews to ensure the three criteria proposed by Damon and colleagues (2003) are met. However, self‐report tools have also been developed to capture beyond‐the‐self purpose consistent with Damon and colleagues's multidimensional definition (e.g., Bronk et al., 2018). These innovations signal the continued valuation of subjective experience under the content perspective. Nonetheless, the “what” of purpose pursuit remains a key element of this approach.

In the present study, we prioritize the sense of purpose perspective. The intentionality and self‐directedness that characterize a sense of purpose are common to both content and sense definitions, allowing us to incorporate both bodies of literature into our review. With conceptual issues in purpose only recently being discussed in the academic literature (e.g., Burrow et al., 2021; Kashdan et al., 2024), such a compromise is necessary to describe purpose development in childhood and adolescence. Nevertheless, in recognition that we may one day find important differences across these conceptualizations, we do our best to state explicitly when the alternative conceptualization, beyond‐the‐self purpose, was the target of investigation in the review that follows.

## Adolescent purpose development and resources for acquisition

As a sense of purpose is thought to be an extension of identity (McKnight & Kashdan, 2009), the topic is often invited into conversations about adolescence, when identity formation is a chief psychosocial task (Erikson, 1959). Underscoring the relevance of adolescence for identity and purpose development, McAdams (2013) suggested it is not until the second decade of life that individuals have the necessary cognitive capacities (e.g., abstract thinking, hypothetical reasoning, internalized regulation) and personal characteristics (e.g., traits, roles, motivations, aspirations) to narratively connect who they have been to who they are and who they are in the process of becoming. It is this authorship capacity—the ability to tell a coherent story about the self—that gives rise to an enduring sense of temporal continuity, personal meaning, and overarching purpose. This developmental account about the emergence of selfhood dovetails nicely with notions of purpose as a late‐developing psychological asset that can help youth begin to turn outward, toward their personal future and potentially the broader world through beyond‐the‐self intentionality (Damon, 2008; Damon et al., 2003).

Yet, despite its recognition as a psychological asset, empirical attempts to map purpose development during adolescence are rare. Purpose appears to increase with age consistent with cognitive maturation and psychosocial changes that happen for youth as they take on new roles, diversify their activities, explore their interests more independently, and expand their social networks. Indeed, when considering beyond‐the‐self purpose development across adolescence and into adulthood, this disposition seems to become more common among adolescents after middle school (e.g., Bronk et al., 2010) and refined during college (Colby, 2020). Such an arc complements the more extensive longitudinal work on adolescent identity development, which has suggested patterns of increasing consolidation over time (e.g., Kroger et al., 2010). But beyond parallel trajectories and theorized ties, there is evidence that identity and purpose act as co‐conspirators in development: purpose may guide identity exploration efforts (Bronk, 2011; Hill & Burrow, 2012), and identity may provide the wherewithal to recognize what purpose‐related information is worth a person's attention when they find it (Burrow & Hill, 2011; Kashdan & McKnight, 2009). If identity and purpose development show similar features and facilitate one another, frameworks describing the resources one can use to establish identity should be useful for understanding the resources one can use to establish purpose. One such framework that offers a way of understanding resources for identity and purpose negotiation is the identity capital model.

For late adolescents and university students, identity capital can be operationalized as successful personal identity (Côté, 1997) and purpose (Burrow & Hill, 2011) acquisition. Reflecting its roots in sociology, identity capital describes the internal compass necessary to transition to adulthood and find one's role in society when society itself increasingly lacks the structure, institutions, and tight‐knit communities to support and shape this process (Côté, 1996, 1997). To develop and maintain identity capital, a person can rely on a number of tangible and intangible resources (Côté, 1996). On the one hand, tangible resources (e.g., privileged socioeconomic background, educational credentials, parent education, linguistic skill) help people access spaces and experiences that enable self‐exploration. On the other hand, intangible resources (e.g., moral reasoning, critical thinking, self‐efficacy, self‐monitoring, self‐exploration, and a sense of personal competence) describe social‐psychological dispositions that help young people navigate nebulous aspects of “late‐modern adult life” (Côté, 1997, p. 578). Several intangible attributes (i.e., ego strength, self‐esteem, internal locus of control, sense of purpose, self‐actualization, ideological commitment) have been used to describe a latent *agentic personality* style that corresponds with the proactive forging of adult identity (i.e., developmental individualization) instead of the uncritical adoption of expectations (i.e., default individualization; Côté, 1997; Côté & Schwartz, 2002; Schwartz et al., 2005). Although a sense of purpose has been implicated in the agentic personality style that can give rise to identity capital, early forms of purpose development (e.g., the task initiative that theoretically develops before a child starts school; Erikson, 1959) and personal agency (McKnight & Kashdan, 2009) may set the stage for the later‐developing higher‐order purpose that also serves as identity capital during the transition to adulthood (Burrow & Hill, 2011).

Given the fluid nature of many late‐ and post‐modern societies, intangible resources from Côté's identity capital model (1996, 1997) are thought to help youth cut through unscripted environments and develop a sense of who they are and where they are going in life. Therefore, many of these attributes should help adolescents make the most of settings that encourage them to chart their own course. When placed in such an environment, we may expect those rich with certain intangible resources to flourish most in terms of purpose development.

## GripTape: An affordance‐rich environment for purpose development

Youth programs have long been recognized as spaces where young people can develop skills, have fulfilling relationships, explore interests, and build character (e.g., Bowers et al., 2015). Headquartered in New York City, GripTape is a remotely administered self‐driven learning program. With the recognition that learning happens everywhere—not just in schools—the “Learning Challenge” is GripTape's flagship program, offering under‐resourced adolescents between the ages of 14 and 19 an opportunity to explore a self‐identified passion for ~10 weeks. In addition to being able to choose almost any topic of personal interest (e.g., software development, animal therapy, criminal justice; Zhu & Burrow, 2022), participants are given a stipend of up to 500 U.S. Dollars (USD); a non‐expert supportive adult (i.e., Champion) to check‐in with them throughout the experience; and total power to choose their direction, decide what targets within their topic are worth pursuing, reconcile obstacles, and progress through the Learning Challenge. Thus, the hallmark of the GripTape program is the centering of youth voice and agency. This often makes Learning Challenge activities of different youth—even those across similar topics—diverse and heterogeneous. Since 2015, GripTape has supported over 3000 adolescents from across the United States. Anecdotal reports tie benefits to participation.

With its bottom‐up orientation to youth learning and provision of resources described above, GripTape may qualify as an “affordance‐rich” environment for purpose development. In other words, by removing barriers to access and inviting youth to explore their aims and interests in almost any way they desire, GripTape may be a setting that offers the tools that youth need to cultivate or strengthen their sense of purpose. Several aspects of GripTape can be tied to theories of how purpose and related constructs develop.

First, Self‐Determination Theory suggests that autonomy, relatedness, and competence are basic psychological needs (Ryan & Deci, 2000). When satisfied, individuals can pursue personal growth and well‐being through upregulated capacities for internal motivation and regulation. Part of the well‐being that can be achieved through psychological needs satisfaction is eudaimonia—a state of living in accordance with the “true self” that is characterized, in part, by a heightened sense of purpose (Huta & Waterman, 2014). With a Champion to connect youth to the program and encourage feelings of belongingness, the opportunity for youth to set their own goals, and the space for youth to build mastery of self‐selected learning content, GripTape may be an environment that supports relatedness, autonomy, and competence. This may clear a path to the sense of purpose that marks eudaimonic flourishing.

Second, theories of adolescent thriving highlight the bidirectional exchange of youth and environments (Benson & Scales, 2009). In short, youth with personal passions, or sparks, can take action to select and shape supportive environments. Then, adults in the environment who build opportunities for spark expression, affirm spark engagement, and apply positive pressure to keep youth moving forward with their sparks create the conditions necessary for thriving. Thriving includes the development of youth purpose. Supporting this view, out‐of‐school time programs with qualities like spark affirmation and high‐quality youth‐adult relationships (e.g., 4‐H) have been held up as developmental contexts for beyond‐the‐self purpose (Arnold & Gagnon, 2019; Scales et al., 2011). Furthermore, in qualitative work with under‐resourced high school students, four factors seemed to contribute most to youths' beyond‐the‐self purpose development as they engaged in a college preparatory program: people (i.e., supportive parents and program mentors), identified passions, propensity, and prosocial motivation (Liang et al., 2017). Thus, by enrolling adolescents with requisite passions, offering dedicated and supportive adults to aid their journey, and providing them with an opportunity to explore their interests, GripTape may be an ideal place for mapping short‐term trends in purpose development.

## The present study

In response to growing concerns about adolescent mental health, recent calls highlight the need to provide young people with more opportunities for exploration and discovery (Fuligni & Galván, 2022). With reason to think GripTape qualifies as an affordance‐rich environment that can respond to these calls, needed are studies that examine how well‐being indicators, like youths' sense of purpose, take shape in this unique context. Using 10 weeks of data, the first aim of this study was to explore how adolescents' sense of purpose changes across the GripTape program. In addition to filling important gaps in the literature about short‐term trends in purpose development, this research will shed light on how promising contexts correspond with the types of outcomes they strive to encourage.

In our second aim, we turned our attention to what attributes predict purpose development. With all youth enrolled in GripTape having access to its resources, we can ask if intangible resources that facilitate self‐exploration assist youth in the self‐directed experience offered by this program. Toward this end, we focused on the predictive capacity of baseline intangible resources most similar to those described by the identity capital model and what Côté and colleagues label “agentic personality” (Schwartz et al., 2005): pre‐program levels of identity exploration and commitment, environmental mastery, agency and the confidence to achieve one's goals through multiple pathways, self‐efficacy, self‐esteem, and tendencies toward self‐reflection and insight. Although largely exploratory and dependent on the trajectories recovered in our first aim, we expected that intangible resources would correspond positively with trajectories reflecting purpose growth across the GripTape experience. In pursuing this second aim, we can conceptually test core assumptions of the identity capital model, as well as begin to understand what resources prepare youth to take advantage of developmental opportunities. Identifying these attributes may create targets for future intervention and inroads for boosting the effectiveness of existing youth programs.

## METHOD

### Participants and procedure

Participants were 321 adolescents (*M*[SD]_age_ = 16.4[1.2] years) enrolled in GripTape. They identified as female (71.0%), male (23.4%), or as a member of a gender minority group (5.5%). In terms of racial‐ethnic background, participating adolescents identified as African American or Black (25.9%), Asian American or Asian (33.3%), Hispanic or Latinx (15.6%), Native American (0.3%), White (13.4%), multiracial (9.7%), or an unlisted racial‐ethnic category (1.2%). As GripTape is remotely administered, adolescents were recruited throughout the United States. Income information was not available to the research team, but all youth qualified as under‐resourced in some capacity (e.g., financially limited, geographically constrained). Indeed, GripTape prefers to enroll youth who would be unlikely to pursue their Learning Challenge topics without their support. Prior to arriving at this analytic sample, data were cleaned of day‐level duplicate entries (first response retained, *m* = 8) and one participant's daily data were removed for their enrollment in an experimental version of GripTape that deviates from the standard administration.

After receiving parent permission and youth assent (or informed consent if applicable), participants were offered 5 USD to take part in a 30‐min baseline survey covering topics across wellness, relationships, learning and motivation, emotions, and self and personality. Although it contained more measures than those described below, the baseline survey included all attributes available to us that we could conceptualize as intangible resources for purpose development (i.e., identity exploration and commitment, environmental mastery, agency, pathway generation, self‐efficacy, self‐esteem, self‐reflection, and insight). After taking the baseline survey, participants embarked on their Learning Challenge. Surveys containing our sense of purpose measure and other assessments were sent to participants each day of the Learning Challenge at 18:00 local time. Participants were given until 23:59 local time to complete the ~5‐min daily survey and were awarded 0.50 USD for each submission. Among those in our analytic sample, Learning Challenges averaged 69.09 days (SD = 15.74; range = 36–126). At the end of the experience, participants were offered an additional 5 USD to take a post‐survey that was largely identical to the baseline survey. All data were collected between January 2021 and September 2022, and procedures were approved by the Cornell University Institutional Review Board.

### Measures

#### Sense of purpose

To index sense of purpose in life, participants used a five‐point Likert scale to respond to the question, “How purposeful do you feel today?” (see Hill et al., 2022). Higher scores corresponded to a stronger sense of daily purpose. To facilitate model convergence, we aggregated the first 70 days of data into 10 weeks and used weekly average sense of purpose to establish purpose trajectories across program enrollment. For participants who extended their Learning Challenges beyond this timeframe (*n* = 71; 22.12%), only the first 10 weeks of data were analyzed. Half of the youth in this subsample completed their Learning Challenge in Week 11 (*n* = 35), meaning that only a few days of data were truncated in most cases. We made this choice to (a) align with GripTape's intended 10‐week program design and (b) mitigate issues stemming from high missingness after the program's preferred endpoint.

We should note that although single‐item assessments have been critiqued in the past for questionable measurement value, the rising popularity of intensive longitudinal research designs has invited interest in the potential of these measures to offset task burden. Several reviews now point to the value of using brief assessments, ranging from adequate conceptual coverage and psychometric properties (e.g., Allen et al., 2022) to practical advantages like increased data quality (Eisele et al., 2022). As reported elsewhere by studies relying on the same data as the present study (Ratner et al., 2024), average daily sense of purpose assessed with our single‐item measure tended to correlate positively with established multi‐item assessments of purpose indexed at baseline: the Life Engagement Test (*r*[319] = .49, *p* < .001; Scheier et al., 2006), the Claremont Purpose Scale for beyond‐the‐self purpose (*r*[319] = .51, *p* < .001; Bronk et al., 2018), and the three‐item purpose in life subscale of the Psychological Well‐being Scale (*r*[316] = .30, *p* < .001; Ryff & Keyes, 1995). Notably, these correlations were at a magnitude similar to other work employing this validation method for single‐item purpose assessments among adolescents (Kiang, 2012).

#### Identity exploration and commitment

Representing the first two baseline intangible resources, identity exploration and commitment were assessed with the exploration in breadth and commitment‐making subscales of the Dimensions of Identity Development Scale (Luyckx et al., 2008). These subscales were chosen because of how closely they align with classic descriptions of Eriksonian identity formation processes (Waterman, 2015). Under this framework, individuals vary in the extent to which they “try out” different ways of identifying the self (exploration) and use these choices to define the self and navigate the world (commitment). Using a five‐point Likert scale with higher scores indicating stronger agreement, participants responded to five items for both identity exploration (e.g., “I am considering a number of different lifestyles that might suit me”) and commitment (e.g., “I have made a choice on what I am going to do with my life”). Cronbach's alpha, a measure of internal consistency or item interrelatedness, for each scale was acceptable (*α*_exploration_ = .86; *α*_commitment_ = .92).

#### Environmental mastery

Environmental mastery is confidence that one can manage the tasks of everyday life. Here, we captured environmental mastery with the corresponding three‐item subscale from Ryff and Keyes' (1995) measure of Psychological Well‐being. Participants rated their agreement with items (e.g., “I am good at managing the responsibilities of daily life”) using a five‐point Likert scale. Cronbach's alpha was reasonable (*α* = .61).

#### Agency and pathways

Agency and pathways were assessed using four‐item subscales of the Adult Hope Scale (Snyder et al., 1991). The agency subscale captures one's sense of having internalized goal‐directed energy (e.g., “I energetically pursue my goals”), whereas the pathways subscale indexes one's capacity for creative and flexible thinking to achieve one's goals (e.g., “I can think of many ways to get the things in life that are important to me”). Participants were asked to rate their agreement with statements on an eight‐point Likert scale. Cronbach's alphas for both the agency (*α* = .76) and pathways (*α* = .67) subscales were adequate.

#### Self‐efficacy

Self‐efficacy was indexed with the 10‐item Generalized Self‐efficacy scale (Schwarzer & Jerusalem, 1995). This scale describes one's beliefs in adaptive coping and internal‐stable self‐attributions for success (e.g., “I am confident that I could deal efficiently with unexpected events”). Participants were asked to rate their agreement with items on a four‐point Likert scale. Cronbach's alpha was good (*α* = .84).

#### Self‐esteem

Self‐esteem, or the tendency to think favorably about the self and make positive self‐evaluations, was assessed with an established single‐item measure (Robins et al., 2001). Participants rated their agreement with this item, “In general, I have high self‐esteem,” on a seven‐point Likert scale.

#### Self‐reflection and insight

Self‐reflection and insight were measured with the 20‐item Self‐reflection and Insight Scale (SRIS; Grant et al., 2002). At the broadest level, this scale captures two processes related to private self‐consciousness: self‐reflection (i.e., how much a person pays attention to their internal thoughts, feelings, and experiences) and insight (i.e., how well a person clearly understands their internal thoughts, feelings, and experiences). Our 12‐item assessment of self‐reflection included items centering on need and engagement (e.g., “I often think about the way I feel about things;” *α* = .90). Insight was measured with eight items and demonstrated good internal consistency (e.g., “I usually have a very clear idea about why I've behaved in a certain way;” *α* = .81). Participants were asked to rate their agreement with all items on a six‐point Likert scale.

Importantly, we decided to exchange several measures on our baseline survey to keep pace with our (and the program's) changing interests throughout the duration of our research‐practitioner partnership. Our partnership extended over four total years: substituting measures as personnel changed and new research questions emerged helped keep the baseline survey at a reasonable length and meet the needs of all parties involved in the broader research effort. The SRIS was removed from our battery during one of these iterations. As such, only a subset of participants had data for self‐reflection and insight (*n* = 192). Analyses with these variables were, therefore, based on a smaller sample.

### Analytic strategy

With Mplus (Version 8.0; Muthén & Muthén, 2017), latent class growth analysis (LCGA) was used to examine whether distinct trajectories of purpose development were present during youths' enrollment in GripTape. Unlike conventional growth modeling approaches that assume all individuals belong to a single population with a similar change pattern, LCGA is a person‐centered technique for identifying latent classes of growth characterized by different initial levels of purpose and rates of change across a given study period (Jung & Wickrama, 2008; Nagin, 2005). The LCGA approach is also the simplest type of growth mixture modeling: it assumes no within‐class variability in growth factors by fixing variance and covariance estimates of intercepts and slopes at zero. With this assumption, LCGA tends to be more computationally straightforward than other growth mixture modeling approaches and identifies latent classes in a clearer manner (Lam et al., 2015). As the first examination of weekly‐level purpose development in out‐of‐school time programming, we saw this study as a foundational test and chose this approach for the simplicity and flexibility it could offer.

In the present study, LCGA unfolded in two stages. We first conducted single‐group unconditional latent growth curve modeling to identify the best‐fitting representation of the average growth pattern in purpose across the entire sample. A no‐growth (intercept‐only) model, a linear growth model, and a quadratic growth model were successively tested. Model fits were evaluated using multiple indices, including the chi‐square test of goodness of fit (*χ* ^2^), comparative fit index (CFI), Tucker–Lewis index (TLI), root mean square error of approximation (RMSEA), and standardized root mean square residual (SRMR). In accordance with common guidance (e.g., Hu & Bentler, 1999; MacCallum et al., 1996; Schumacker & Lomax, 2004), CFI and TLI values ≥.95 and SRMR and RMSEA values ≤.08 were used as thresholds to indicate a good model fit. Additionally, a likelihood ratio test (LRT) was performed to directly compare the fits of the three models. The best‐fitted model was chosen as the preliminary model for subsequent mixture modeling (Ram & Grimm, 2009).

Following the selection of the preliminary model, LCGA was performed following the three‐step procedure recommended by Asparouhov and Muthén (2014). First, starting from a 2‐group solution, latent class models with varying numbers of sub‐trajectories were specified. The optimal number of groups was determined by examining both statistical evidence and substantive interpretations (Johnson, 2021; Ram & Grimm, 2009). Statistical evidence considered in this procedure included the Akaike information criterion (AIC), Bayesian information criterion (BIC), sample‐size‐adjusted BIC (SABIC), Vuong‐LoMendell‐Rubin LRT (VLMR‐LRT), LoMendel‐Rubin LRT (LMR‐LRT), entropy index, and the smallest class size (SCS; see Johnson, 2021; Jung & Wickrama, 2008; Lo et al., 2001; Ram & Grimm, 2009). For AIC, BIC, and SABIC, lower values tend to indicate a better model fit. In contrast, VLMR‐LRT and LMR‐LRT compare neighboring class models. A significant *p*‐value in these LRTs indicates the *k*‐class model significantly fits better than the model with *k*‐1 classes. Next, entropy scores (range 0–1) were used to assess the accuracy of latent class membership assignments. According to Ram and Grimm (2009), an entropy value greater than 0.8 indicates good classification accuracy. The last index, the SCS, serves to prevent overextraction (Johnson, 2021). While there is no established cutoff for the SCS, most ensure at least 5% of the sample appears in each class (e.g., Nylund et al., 2007).

After determining the most appropriate number of latent classes to describe purpose development, the second step was to assign individuals to the most likely trajectory classes. In step 3, intangible resources were added to the model as covariates to investigate their association with purpose trajectory class memberships. This was accomplished through logistic regression (multinomial logistic regression if *k* = 3+). Guided by Asparouhov and Muthén (2014), the above three steps of latent class specification, class assignment, and class membership prediction were executed automatically using the R3STEP option in Mplus.

Across the 10 weeks of GripTape enrollment, the percentage of missing data by case varied from 2.50% to 65.73%. Normed chi‐square (*χ* ^2^/df = 1.38) fell within an acceptable range (1.0–5.0; Schumacker & Lomax, 2004), suggesting that the data were likely missing at random. Therefore, in the step of trajectory identification, missing values were addressed using maximum likelihood estimation with robust standard errors. For baseline intangible resources, missing data ranged from 0.03% to 0.09%. With the R3STEP option in Mplus, listwise deletion was applied in the analysis of trajectory prediction, resulting in the removal of three participants. All scales were maintained on their original, raw scale for analysis.

## RESULTS

### Identification of purpose trajectories

First, we conducted unconditional growth curve modeling and selected the preliminary growth model by comparing the fits of three candidate models (intercept‐only, linear, quadratic; see Table 1). A linear growth model fit the data significantly better than an intercept‐only model (Δ*χ* ^2^ = 96.28, *p* < .001), but a quadratic growth model significantly improved fit over the linear growth model (Δ*χ* ^2^ = 25.14, *p* < .001). Therefore, the quadratic growth model—with an intercept of 3.22 (SE = .045, *p* < .001), a non‐significant linear slope of .03 (SE = .018, *p* = .086), and a non‐significant quadratic slope of −.0004 (SE = .002, *p* = .832)—stood out as the best representation of the growth pattern across the entire sample. Thus, in addition to slight growth and curvature necessary to optimize fit, this model suggested that youth generally had a high purpose starting level and stable developmental trend across 10 weeks of GripTape.

**TABLE 1:** Comparison of unconditional growth curve models for preliminary model fit.

Model	*χ* ^2^ (*df*)	CFI	TLI	RMSEA	SRMR
Intercept‐only	200.34 (53)	.90	.91	.09	.12
Linear	104.06 (50)	.96	.97	.06	.09
Quadratic	78.93 (46)	.98	.98	.05	.05

Abbreviations: CFI, comparative fit index, RMSEA, root mean square error of approximation; SRMR, standardized root mean square residual; TLI, Tucker–Lewis index.

Upon selecting the preliminary growth model, LCGA models with a varying number of latent classes were specified. Model fit indices are presented in Table 2. The VLMR‐LRT and LMR‐LRT indices revealed that the 2‐class solution exhibited a significantly better fit than the 1‐class solution, but no significant difference was found between the 3‐class and 2‐class solutions. However, literature on growth mixture modeling (e.g., Johnson, 2021; Ram & Grimm, 2009) emphasizes that the model selection step should not rely solely on a single indicator but should consider a range of statistical indices along with substantive justifications. Consequently, we extended our model testing beyond the 3‐class solution. As the number of classes increased, there was a consistent decrease in AIC, BIC, and SABIC values. Entropy values remained above 0.8, suggesting good classification accuracy across all models tested. The VLMR‐LRT and LMR‐LRT indicated an improved model fit for the 4‐class solution compared to the 3‐class solution, but no significant difference was observed between the 4‐class and 5‐class solutions. Additionally, for the 5‐class solution, the smallest sample size constituted only 3.12% of the entire sample. Hence, we concluded the model testing process with the 5‐class solution.

**TABLE 2:** Fit indices of 1‐class to 5‐class models.

Group (*k*)	Log‐likelihood	AIC	BIC	SABIC	VLMR‐LRT	LMR‐LRT	Entropy	SCS N%
1	−3032.84	6091.67	6140.70	6099.47	N/A	N/A	N/A	N/A
**2**	**−2621.50**	**5277.00**	**5341.11**	**5287.19**	**<0.001**	**<0.001**	**0.81**	**45.48**
3	−2478.07	4998.14	5077.34	5010.73	0.23	0.23	0.81	18.69
4	−2385.60	4821.19	4915.48	4836.18	<0.001	<0.001	0.80	11.84
5	−2363.03	4784.07	4893.44	4801.46	0.40	0.41	0.80	3.12

Abbreviations: AIC, Akaike information criterion; SABIC, sample‐size adjusted BIC; BIC, Bayesian information criterion; LMR‐LRT, LoMendell‐Rubin likelihood ratio test; SCS, smallest class size, where the percentage represents the proportion of the sample falling into the smallest class in the solution; VLMR‐LRT, Vuong‐LoMendell‐Rubin likelihood ratio test. Bolded row highlights the selected solution.

Given the above considerations, both the 2‐class and the 4‐class models could have been selected as appropriate solutions. However, from a statistical standpoint, the 2‐class model yielded a slightly higher entropy value; offered greater power through its more balanced class size proportions; and was more parsimonious, which could lend itself to replicability. Furthermore, from a practical standpoint, the 4‐class solution was largely redundant with the 2‐class solution, suggesting two more small groups (<15% of the sample each) that were more exaggerated versions of the two groups recovered in the 2‐class solution. This would make it difficult, for example, to offer targeted support to participants in each specific subgroup. As such, we felt the 4‐class solution offered little substantive value beyond the 2‐class solution. Thus, the 2‐class model was chosen as the optimal solution and carried forward with analysis.

**FIGURE 1:**
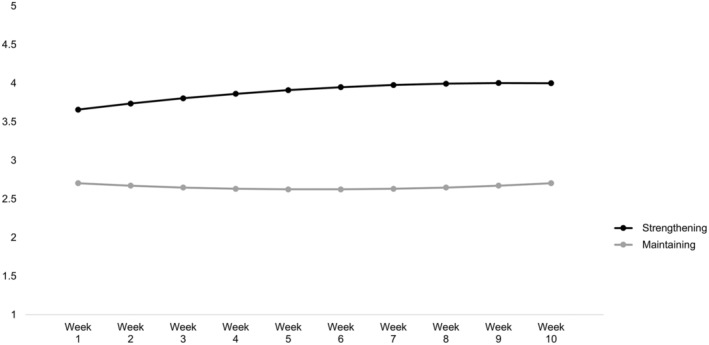
Trajectories of two latent purpose development classes.

Figure 1 visualizes the two trajectories of purpose development implied by the 2‐class model, with corresponding parameter estimates presented in Table 3. The first class, named *Strengthening* (*n* = 175; 54.52%), comprised participants who started with a high level of purpose and showed relatively continuous linear growth over the 10‐week observation period. The second class, labeled *Maintaining* (*n* = 146; 45.48%), comprised participants with moderate initial purpose and a stable developmental trend.

**TABLE 3:** Parameter estimates for the selected two‐class model.

	Parameter	Estimate	SE
Strengthening	Intercept	3.66*	.07
	Linear slope	0.08*	.03
	Quadratic slope	−0.01	.00
Maintaining	Intercept	2.70*	.06
	Linear slope	−0.04	.03
	Quadratic slope	0.00	.00

* *p* ≤ .001.

Finally, we used analysis of variance and chi‐square tests to explore the characteristics of the two classes. However, the two classes were not significantly different in terms of race (*χ* ^2^[6] = 3.17, *p* = .788), gender identity (*χ* ^2^[5] = 10.00, *p* = .075), or age (*F*[1, 279] = 0.83, *p* = .363). We also found no systematic differences between groups in terms of the number of participants who completed their Learning Challenge within the first 10 weeks and those who required additional time (*χ* ^2^[1] = 0.11, *p* = .701).

### Predicting purpose trajectories from intangible identity capital resources

With trajectories established, we turned to our second aim: investigating predictors of purpose growth across the GripTape experience. All descriptive statistics and intercorrelations for the baseline intangible resources are listed in Table 4. In terms of notable trends, all associations between predictors were positive if statistically significant, no associations exceeded a moderate correlation of *r*(317) = .59, *p* < .001 (self‐efficacy and pathways), and we failed to find cross‐sectional associations between identity exploration, self‐reflection, and other intangible resources. In standalone logistic regression models—analogous to the third step of the 3‐step procedure we focused on below—all intangible resource predictors demonstrated a variance inflation factor (VIF) under 1.98. Therefore, although the intangible resources were moderately correlated, we failed to find evidence of multicollinearity (i.e., VIF ≥10.0).

**TABLE 4:** Intercorrelation matrix and descriptive statistics of intangible resource predictors.

Variable	1	2	3	4	5	6	7	8	9
1. Identity exploration	—								
2. Identity commitment	.09	—							
3. Environmental mastery	−.05	.30*	—						
4. Agency	.04	.37*	.54*	—					
5. Pathways	.00	.24*	.39*	.56*	—				
6. Self‐efficacy	−.04	.28*	.48*	.58*	.59*	—			
7. Self‐esteem	.01	.26*	.47*	.43*	.25*	.29*	—		
8. Self‐reflection	.21	.00	−.13	.12	.20	.06	−.07	—	
9. Insight	.01	.19	.48*	.43*	.32*	.30*	.40*	.08	—
*M*	4.1	3.7	3.2	6.2	6.2	3.1	4.3	4.7	3.8
SD	0.7	0.9	0.8	1.1	1.0	0.4	1.7	0.8	0.9

*Note*: *p*‐Value adjustment method: Holm (1979).

* *p* ≤ .001.

To identify predictors of purpose trajectory, class membership (*Strengthening* [1] or *Maintaining* [0]) served as a categorical dependent variable, and intangible resources were added simultaneously as covariates to the three‐step model. With only two trajectory classes, the three‐step model defaulted to logistic regression (Table 5). Agency was the only significant and unique predictor of purpose trajectory class membership. Specifically, for each unit increase in a youth's baseline agency score, the likelihood of being categorized as *Strengthening* was about 1.6 times higher than that of being categorized as *Maintaining*. Thus, high agency was associated with youth who tended to increase on sense of purpose during GripTape enrollment.

**TABLE 5:** The association between intangible identity capital resources and purpose trajectory membership.

Variables	Coef	SE Coef	OR
Identity exploration	.33	.22	1.40
Identity commitment	.21	.18	1.23
Environmental mastery	.56	.29	1.76
Self‐efficacy	.61	.49	1.85
Agency	.46	.22	1.59*
Pathways	.01	.23	1.01
Self‐esteem	.15	.11	1.16
Self‐reflection^a^	.05	.25	1.06
Insight^a^	−.37	.25	0.69

Abbreviations: Coef, coefficient; OR, odds ratio.

^a^ Because of listwise deletion, coefficients for self‐reflection and insight were based on a second analysis using only a subset of participants (*n* = 192). The effects of all other predictors were controlled in this analysis for consistency. All other coefficients are based on analysis with the full sample with self‐reflection and insight covariates excluded.

* *p* ≤ .05.

## DISCUSSION

Theory and evidence point to how a sense of purpose *should* respond to affordance‐rich environments (e.g., Arnold & Gagnon, 2019; Benson & Scales, 2009; Kashdan & McKnight, 2009), but researchers and practitioners have little idea of how purpose may change over time in these contexts. Rarer still are investigations of the resources young people need to develop a greater sense of purpose when such opportunities are provided. This study makes headway on both missing pieces. First, we asked how a sense of purpose may change during GripTape, an out‐of‐school time program that provides resources and encourages youth to engage with their passions through self‐driven learning for ~10 weeks. Then, we investigated individual differences that may help youth capitalize on the self‐exploration opportunity that GripTape offers. With an eye toward how results may inform future research and practice, we review each of our main findings in turn.

On average, youth reported a relatively high and stable sense of purpose during the GripTape program. Although slight linear growth and deceleration statistically optimized the average growth solution, these values were not significant. However, the lack of evidence for change in sense of purpose across GripTape could be due to a genuine lack of change *or* the presence of underlying heterogeneity in purpose development across youth. Our results suggest the latter is the case, with two distinct classes of purpose trajectories emerging from our investigation.

In the first class, youth enrolled in GripTape with high initial purpose and reported increases in their sense of purpose across the experience. These youth, who represented a plurality of the sample, were labeled as *Strengthening*. In the second class, youth reported an initial sense of purpose around the scale midpoint and maintained this level throughout enrollment. These youth, in contrast, were labeled as *Maintaining*. Previous research has rarely examined change during out‐of‐school time programming with the granularity of the present study, but these findings are consistent with work suggesting that carefully designed youth programs tend to be associated with character development (e.g., Arnold & Gagnon, 2019; Bowers et al., 2015). With respect to sense of purpose in particular, some growth could be due to the opportunity GripTape provides to engage with self‐concordant goals for a concentrated period of time (see theory in Hill et al., 2023; Kashdan & McKnight, 2009). Nevertheless, with purpose growth only happening for some youth within GripTape, investigating what resources help youth gain from this experience—perhaps as a member of the *Strengthening* group—is a critical next step for understanding how youth can be prepared to encounter similar developmental opportunities.

This next question about what prepares youth to capitalize on GripTape in terms of purpose development leads to our second main finding. Out of several attributes measured among youth at baseline, only agency emerged as a unique and significant predictor of purpose trajectory class membership. For each unit increase in baseline agency, youth were 1.6× more likely to be classified within the purpose *Strengthening* group than the *Maintaining* group. This suggests that having internal, goal‐directed energy may have been key to using the GripTape experience to fortify one's overarching sense of purpose.

Such findings align with the identity capital model (Côté, 1997; Côté & Schwartz, 2002), which posits that an agentic personality may help young people cut through unscripted environments and cultivate higher‐order aspects of themselves critical to finding a role in adult society (Schwartz et al., 2005). For this reason, perhaps agency can serve as a target for future intervention: empowering youth to pursue their goals may help them find and make the most of blank‐canvas opportunities like GripTape and other theoretically favorable environments. Although intervention could take many forms (see Banerjee et al., 2024), one promising avenue is to give youth early and frequent opportunities to make a tangible impact on their environment. Even if more directed, these experiences have been argued to lay the foundation for autonomous and agentic functioning that may benefit youth later in unscripted contexts (e.g., Fuligni, 2019; Fuligni & Galván, 2022).

Still, it is worth noting that the identity capital model proposes *many* intangible resources for identity and purpose development (Burrow & Hill, 2011; Côté, 1997). Several of these resources were investigated in the present study, yet only agency emerged as a significant predictor of purpose trajectory class membership. As such, while our second group of results are certainly aligned with core notions of the identity capital model, there are aspects of the model that remain in question. For example, whereas ideological commitment (identity commitment in the present study) is a component of the agentic personality style nominated by Schwartz et al. (2005), we failed to find evidence that it distinguished between purpose *Strengthening* and *Maintaining* groups. One potential explanation for this divergence could be due to the level of ideological commitment that we assessed. Commitment making is an initial stage of identity formation, most closely aligned with early operationalizations of identity commitment (Waterman, 2015). However, identification with commitment—a deeper, evaluative component of identity development that occurs when self‐concept becomes tied to one's ideological choices (Luyckx et al., 2008)—may be closer to the type of ideological commitment that Schwartz et al. (2005) suggested can help one cultivate identity capital. As such, we warn against those quick to write off ideological commitment as a resource for identity and purpose consolidation in youth programs based on our study. Future research is necessary to test this potential explanation surrounding the “level” of ideological commitment before such claims could be made about its usefulness as an intangible resource.

### Limitations and other future directions

This study presents one of the finest examinations of purpose development within an out‐of‐school time program to date. However, despite what it can offer the field, some limitations of this work should be noted. Nonetheless, these limitations can act as springboards for future research and practical application.

First, we see obvious questions surrounding what qualifies as an “affordance‐rich” environment for purpose development. While we stand by our claim that GripTape has traditional affordances that youth are likely to benefit from, what makes an environment “affordance‐rich” may differ across people. For example, are there environments outside of GripTape where those in the *Maintaining* group are more likely to flourish? Youth in the *Maintaining* group may have needed a program with more scaffolding or direction to create a developmental opportunity: it is possible youth in this group were pursuing Learning Challenge topics that were strictly hedonic and not congruent with their purpose. Indeed, “the presence of a purpose can be defined as a passionate interest, [but] not all interests or passions can be construed as a purpose” (Kashdan & McKnight, 2009, pp. 306–307).

Along this same line of thinking, do those in the *Maintaining* group need additional resources to take advantage of GripTape? Perhaps those in the *Maintaining* group were missing important external support (e.g., family endorsement, community outlets, economic flexibility) necessary to generalize the purposefulness they felt during their Learning Challenge, allowing it to grow during the program. Congruence and feasibility—the degree to which a purpose fits within one's local ecology and can easily be pursued—have been argued to underpin purpose development (Burrow et al., 2021). Without external support, any purpose development that occurred during GripTape may have dropped off quickly, resulting in the two distinguishable groups we recovered. The current study is limited by both the environments we can examine for purpose development and the resources we can test as predictors of developmental success within GripTape. Future work may investigate a broader range of programs where purpose development could be taking place for youth, as well as consider a suite of other resources youth could use to profit from the opportunity GripTape offers. Efforts here may help the field better understand what youth need to capitalize on spaces with traditional notions of affordance, as well as create a more inclusive definition of “affordance‐rich” programming.

Second, future research may ask if agency is associated with purpose (or other character) growth in other youth‐facing programs. This may begin to answer an initial set of questions surrounding the generalizability of our findings. While agency may be an intangible resource that helps youth prepare for GripTape and similar experiences, it may not be universally resourceful in all environments. Before interventions attempt to boost youth agency prior to out‐of‐school time programming, direct replication within and outside of GripTape should be conducted to help define the scope of our findings.

Third, it would be prudent to note a few potential issues related to our sampling strategy and measurement selection so they can be addressed by future research. Related to sampling, youth across both the *Strengthening* and *Maintaining* groups averaged relatively high levels of purpose in Week 1 of the GripTape program. This is likely a reflection of who the program admits: youth must articulate their learning goals in their application, applications may be selected based on clarity, and goal orientation and clarity are likely to correlate with purpose at baseline. There is debate in the literature about the atypicality of purpose among adolescents, with beyond‐the‐self notions of purpose appearing relatively rare (Bronk et al., 2010) and sense of purpose notions appearing relatively common (Hill & Burrow, 2021). Still, conceptual issues aside, it is reasonable to wonder if our *Strengthening* and *Maintaining* groups would be recovered in a more representative sample of youth entering GripTape. The trajectory of purpose development for low‐purpose youth within GripTape and like‐programs remains unknown, but future work in this space (e.g., if GripTape enrollment was randomized) could shed light on the utility of these programs for purposes that must develop “from scratch.”

Related to measurement, future work would also benefit from efforts to establish a single‐item purpose assessment for intensive longitudinal research designs. Although we provide circumstantial evidence for the utility of our single‐item assessment (i.e., prior research using the measure, correlations with established multi‐item scales, qualitative evidence of youths' “purpose” understanding), work remains to establish other important aspects of daily assessment, like discriminant validity. This is especially critical given recent empirical research demonstrating a happiness factor, or “h‐factor,” among well‐being constructs belonging to the purpose nomological net (Bjørndal et al., 2023). This h‐factor is a generalized, second‐order factor capturing shared variance among neighboring constructs, mirroring concepts like the g‐factor in intelligence testing and the p‐factor in psychopathology. With ongoing research in this area, we may increase the robustness and trustworthiness of single‐item assessments and open the door for close examinations of purpose fluctuation over time.

Fourth and finally, we must take caution in assuming that the purpose development observed in this study during GripTape enrollment is causally associated with program participation. To draw these inferences, it would be necessary to either track youth development before and after the GripTape experience to see how trends may change leading up to, and after, program participation, or recruit a sample of non‐GripTape youth and compare developmental trends during the same period. Although this study is built on literature suggesting that out‐of‐school time programs have benefits for youth development (e.g., Bowers et al., 2015), the pairing of purpose “activation” within the GripTape program remains a question that only comparative studies can answer. The present study may motivate this work to take place.

## CONCLUSION

There are a number of theories surrounding how purpose develops (Hill et al., 2023; Kashdan & McKnight, 2009) and how specific environments can be built to help youth cultivate it (e.g., Benson & Scales, 2009). However, despite this attention, there remains little evidence to show the developmental arc of purpose in affordance‐rich conditions. Furthermore, existing research fails to illuminate *what* helps purpose develop under these favorable circumstances. In a program that helps adolescents engage with their self‐identified passions for 10 weeks, our work shows that highly agentic youth tend to strengthen their sense of purpose during enrollment. Targeting agency in the future—to help youth prepare for GripTape and like‐environments—may make this growth more widespread. In establishing these patterns, this study sheds light on important missing parts of the youth purpose tapestry. Our results also lay the foundation for future work to build upon, so we may one day recognize a wider range of purpose‐supportive environments.

## FUNDING INFORMATION

This project has been made possible in part by a grant from the Chan Zuckerberg Initiative DAF (No. 136823), an advised fund of Silicon Valley Community Foundation.

REFERENCES

Allen, M. S.
, 
Iliescu, D.
, & 
Greiff, S.
 (2022). Single item measures in psychological science: A call to action. European Journal of Psychological Assessment, 38, 1–5. 10.1027/1015-5759/a000699


Arnold, M. E.
, & 
Gagnon, R. J.
 (2019). Illuminating the process of youth development: The mediating effect of thriving on youth development program outcomes. Journal of Human Sciences and Extension, 7, 24–51. 10.54718/GHUP2927


Asparouhov, T.
, & 
Muthén, B.
 (2014). Auxiliary variables in mixture modeling: Three‐step approaches using Mplus. Structural Equation Modeling, 21, 329–341. 10.1080/10705511.2014.915181


Banerjee, S.
, 
Grüne‐Yanoff, T.
, 
John, P.
, & 
Moseley, A.
 (2024). It's time we put agency into behavioural public policy. Behavioural Public Policy, 8, 789–806. 10.1017/bpp.2024.6


Benson, P. L.
, & 
Scales, P. C.
 (2009). The definition and preliminary measurement of thriving in adolescence. The Journal of Positive Psychology, 4, 85–104. 10.1080/17439760802399240


Bjørndal, L. D.
, 
Nes, R. B.
, 
Czajkowski, N.
, & 
Røysamb, E.
 (2023). The structure of well‐being: A single underlying factor with genetic and environmental influences. Quality of Life Research, 32, 2805–2816. 10.1007/s11136-023-03437-7
37209357
PMC10199429


Bowers, E. P.

, 

Geldhof, G. J.

, 

Johnson, S. K.

, 

Hilliard, L. J.

, 

Hershberg, R. M.

, 

Lerner, J. V.

, & 

Lerner, R. M.

 (Eds.). (2015). Promoting positive youth development: Lessons from the 4‐H study. Springer International Publishing. 10.1007/978-3-319-17166-1


Bronk, K. C.
 (2011). The role of purpose in life in healthy identity formation: A grounded model. New Directions for Youth Development, 2011, 31–44. 10.1002/yd.426
22275277

Bronk, K. C.
, 
Finch, H.
, & 
Talib, T.
 (2010). Purpose in life among high ability adolescents. High Ability Studies, 21, 133–145. 10.1080/13598139.2010.525339


Bronk, K. C.
, 
Riches, B. R.
, & 
Mangan, S. A.
 (2018). Claremont Purpose Scale: A measure that assesses the three dimensions of purpose among adolescents. Research in Human Development, 15, 101–117. 10.1080/15427609.2018.1441577


Burrow, A. L.
, 
Agans, J. P.
, 
Jeon, H. J.
, & 
Creim, M.
 (2021). Are all purposes worth having? Integrating content and strength in purpose research. Human Development, 65, 100–112. 10.1159/000515176


Burrow, A. L.
, & 
Hill, P. L.
 (2011). Purpose as a form of identity capital for positive youth adjustment. Developmental Psychology, 47, 1196–1206. 10.1037/a0023818
21574703


Colby, A.
 (2020). Purpose as a unifying goal for higher education. Journal of College and Character, 21, 21–29. 10.1080/2194587X.2019.1696829


Côté, J. E.
 (1996). Sociological perspectives on identity formation: The culture‐identity link and identity capital. Journal of Adolescence, 19, 417–428. 10.1006/jado.1996.0040
9245295


Côté, J. E.
 (1997). An empirical test of the identity capital model. Journal of Adolescence, 20, 577–597. 10.1006/jado.1997.0111
9368134


Côté, J. E.
, & 
Schwartz, S. J.
 (2002). Comparing psychological and sociological approaches to identity: Identity status, identity capital, and the individualization process. Journal of Adolescence, 25, 571–586. 10.1006/jado.2002.0511
12490176


Damon, W.
 (2008). The path to purpose: How young people find their calling in life. Free Press.

Damon, W.
, 
Menon, J.
, & 
Bronk, K. C.
 (2003). The development of purpose during adolescence. Applied Developmental Science, 7, 119–128. 10.1207/S1532480XADS0703_2


Eisele, G.
, 
Vachon, H.
, 
Lafit, G.
, 
Kuppens, P.
, 
Houben, M.
, 
Myin‐Germeys, I.
, & 
Viechtbauer, W.
 (2022). The effects of sampling frequency and questionnaire length on perceived burden, compliance, and careless responding in experience sampling data in a student population. Assessment, 29, 136–151. 10.1177/1073191120957102
32909448


Erikson, E. H.
 (1959). Identity and the life cycle. International Universities Press.

Fuligni, A. J.
 (2019). The need to contribute during adolescence. Perspectives on Psychological Science, 14, 331–343. 10.1177/1745691618805437
30562473
PMC6497551

Fuligni, A. J.
, & 
Galván, A.
 (2022). Young people need experiences that boost their mental health. Nature, 610, 253–256. 10.1038/d41586-022-03172-y
36216911


Grant, A. M.
, 
Franklin, J.
, & 
Langford, P.
 (2002). The self‐reflection and insight scale: A new measure of private self‐consciousness. Social Behavior and Personality: An International Journal, 30, 821–834. 10.2224/sbp.2002.30.8.821


Hill, P. L.
, & 
Burrow, A. L.
 (2012). Viewing purpose through an Eriksonian lens. Identity: An International Journal of Theory and Research, 12, 74–91. 10.1080/15283488.2012.632394


Hill, P. L.
, & 
Burrow, A. L.
 (2021). Why youth are more purposeful than we think. Child Development Perspectives, 15, 281–286. 10.1111/cdep.12432


Hill, P. L.
, 
Burrow, A. L.
, 
O'Dell, A. C.
, & 
Thornton, M. A.
 (2010). Classifying adolescents' conceptions of purpose in life. The Journal of Positive Psychology, 5, 466–473. 10.1080/17439760.2010.534488


Hill, P. L.
, 
Klaiber, P.
, 
Burrow, A. L.
, 
DeLongis, A.
, & 
Sin, N. L.
 (2022). Purposefulness and daily life in a pandemic: Predicting daily affect and physical symptoms during the first weeks of the COVID‐19 response. Psychology & Health, 37, 985–1001. 10.1080/08870446.2021.1914838
33974470


Hill, P. L.
, 
Pfund, G. N.
, & 
Allemand, M.
 (2023). The PATHS to purpose: A new framework toward understanding purpose development. Current Directions in Psychological Science, 32, 105–110. 10.1177/09637214221128019


Holm, S.
 (1979). A simple sequentially rejective multiple test procedure. Scandinavian Journal of Statistics, 6, 65–70. 10.2307/4615733


Hu, L.
, & 
Bentler, P. M.
 (1999). Cutoff criteria for fit indexes in covariance structure analysis: Conventional criteria versus new alternatives. Structural Equation Modeling, 6, 1–55. 10.1080/10705519909540118


Huta, V.
, & 
Waterman, A. S.
 (2014). Eudaimonia and its distinction from hedonia: Developing a classification and terminology for understanding conceptual and operational definitions. Journal of Happiness Studies, 15, 1425–1456. 10.1007/s10902-013-9485-0


Johnson, S. K.
 (2021). Latent profile transition analyses and growth mixture models: A very non‐technical guide for researchers in child and adolescent development. New Directions for Child and Adolescent Development, 2021, 111–139. 10.1002/cad.20398
33634554

Jung, T.
, & 
Wickrama, K. A. S.
 (2008). An introduction to latent class growth analysis and growth mixture modeling. Social and Personality Psychology Compass, 2, 302–317. 10.1111/j.1751-9004.2007.00054.x


Kashdan, T. B.
, 
Goodman, F. R.
, 
McKnight, P. E.
, 
Brown, B.
, & 
Rum, R.
 (2024). Purpose in life: A resolution on the definition, conceptual model, and optimal measurement. American Psychologist, 79, 838–853. 10.1037/amp0001223
37982782


Kashdan, T. B.
, & 
McKnight, P. E.
 (2009). Origins of purpose in life: Refining our understanding of a life well‐lived. Psychological Topics, 18, 303–316.

Kiang, L.
 (2012). Deriving daily purpose through daily events and role fulfillment among Asian American youth. Journal of Research on Adolescence, 22, 185–198. 10.1111/j.1532-7795.2011.00767.x


Kroger, J.
, 
Martinussen, M.
, & 
Marcia, J. E.
 (2010). Identity status change during adolescence and young adulthood: A meta‐analysis. Journal of Adolescence, 33, 683–698. 10.1016/j.adolescence.2009.11.002
20004962


Lam, S.‐F.
, 
Law, W.
, 
Chan, C.‐K.
, 
Wong, B. P. H.
, & 
Zhang, X.
 (2015). A latent class growth analysis of school bullying and its social context: The self‐determination theory perspective. School Psychology Quarterly, 30, 75–90. 10.1037/spq0000067
24884451


Lewis, N. A.
 (2020). Purpose in life as a guiding framework for goal engagement and motivation. Social and Personality Psychology Compass, 14, 1–11. 10.1111/spc3.12567


Liang, B.
, 
White, A.
, 
Mousseau, A. M. D.
, 
Hasse, A.
, 
Knight, L.
, 
Berado, D.
, & 
Lund, T. J.
 (2017). The four P's of purpose among college bound students: People, propensity, passion, prosocial benefits. The Journal of Positive Psychology, 12, 281–294. 10.1080/17439760.2016.1225118


Lo, Y.
, 
Mendell, N. R.
, & 
Rubin, D. B.
 (2001). Testing the number of components in a normal mixture. Biometrika, 88, 767–778. 10.1093/biomet/88.3.767


Luyckx, K.
, 
Schwartz, S. J.
, 
Berzonsky, M. D.
, 
Soenens, B.
, 
Vansteenkiste, M.
, 
Smits, I.
, & 
Goossens, L.
 (2008). Capturing ruminative exploration: Extending the four‐dimensional model of identity formation in late adolescence. Journal of Research in Personality, 42, 58–82. 10.1016/j.jrp.2007.04.004


MacCallum, R. C.
, 
Browne, M. W.
, & 
Sugawara, H. M.
 (1996). Power analysis and determination of sample size for covariance structure modeling. Psychological Methods, 1, 130–149. 10.1037/1082-989X.1.2.130


McAdams, D. P.
 (2013). The psychological self as actor, agent, and author. Perspectives on Psychological Science, 8, 272–295. 10.1177/1745691612464657
26172971


McKnight, P. E.
, & 
Kashdan, T. B.
 (2009). Purpose in life as a system that creates and sustains health and well‐being: An integrative, testable theory. Review of General Psychology, 13, 242–251. 10.1037/a0017152


Muthén, L. K.
, & 
Muthén, B. O.
 (2017). Mplus user's guide (8th ed.). Muthén & Muthén.

Nagin, D. S.
 (2005). Group‐based modeling of development. Harvard University Press. 10.4159/9780674041318


Nylund, K. L.
, 
Asparouhov, T.
, & 
Muthén, B.
 (2007). Deciding on the number of classes in latent class analysis and growth mixture modeling: A monte carlo simulation study. Structural Equation Modeling, 14, 535–569. 10.1080/10705510701575396


Ram, N.
, & 
Grimm, K. J.
 (2009). Growth mixture modeling: A method for identifying differences in longitudinal change among unobserved groups. International Journal of Behavioral Development, 33, 565–576. 10.1177/0165025409343765
23885133
PMC3718544

Ratner, K.
, 
Burrow, A. L.
, 
Burd, K. A.
, & 
Hill, P. L.
 (2021). On the conflation of purpose and meaning in life: A qualitative study of high school and college student conceptions. Applied Developmental Science, 25, 364–384. 10.1080/10888691.2019.1659140


Ratner, K.
, 
Gladstone, J. R.
, 
Zhu, G.
, 
Li, Q.
, 
Estevez, M.
, & 
Burrow, A. L.
 (2024). Purpose and goal pursuit as a self‐sustaining system: Evidence of daily within‐person reciprocity among adolescents in self‐driven learning. Journal of Personality, 92, 1556–1570. 10.1111/jopy.12911
38108114


Robins, R.
, 
Hendin, H.
, & 
Trzesniewski, K.
 (2001). Measuring global self‐esteem: Construct validation of a single‐item measure and the Rosenberg Self‐Esteem Scale. Personality and Social Psychology Bulletin, 27, 151–161. 10.1177/0146167201272002


Ryan, R. M.
, & 
Deci, E. L.
 (2000). Self‐determination theory and the facilitation of intrinsic motivation, social development, and well‐being. American Psychologist, 55, 68–78. 10.1037/0003-066X.55.1.68
11392867


Ryff, C. D.
 (1989). Happiness is everything, or is it? Explorations on the meaning of psychological well‐being. Journal of Personality and Social Psychology, 57, 1069–1081. 10.1037/0022-3514.57.6.1069


Ryff, C. D.
, & 
Keyes, C. L. M.
 (1995). The structure of psychological well‐being revisited. Journal of Personality and Social Psychology, 69, 719–727. 10.1037/0022-3514.69.4.719
7473027


Scales, P. C.
, 
Benson, P. L.
, 
Leffert, N.
, & 
Blyth, D. A.
 (2000). Contribution of developmental assets to the prediction of thriving among adolescents. Applied Developmental Science, 4, 27–46. 10.1207/S1532480XADS0401_3


Scales, P. C.
, 
Benson, P. L.
, & 
Roehlkepartain, E. C.
 (2011). Adolescent thriving: The role of sparks, relationships, and empowerment. Journal of Youth and Adolescence, 40, 263–277. 10.1007/s10964-010-9578-6
20680424


Scheier, M. F.
, 
Wrosch, C.
, 
Baum, A.
, 
Cohen, S.
, 
Martire, L. M.
, 
Matthews, K. A.
, 
Schulz, R.
, & 
Zdaniuk, B.
 (2006). The Life Engagement Test: Assessing purpose in life. Journal of Behavioral Medicine, 29, 291–298. 10.1007/s10865-005-9044-1
16565785


Schumacker, R. E.
, & 
Lomax, R. G.
 (2004). A beginner's guide to structural equation modeling (2nd ed.). Lawrence Erlbaum Associates Publishers.

Schwartz, S. J.
, 
Côté, J. E.
, & 
Arnett, J. J.
 (2005). Identity and agency in emerging adulthood: Two developmental routes in the individualization process. Youth & Society, 37, 201–229. 10.1177/0044118X05275965


Schwarzer, R.
, & 
Jerusalem, M.
 (1995). Generalized self‐efficacy scale. In 

J.
Weinman

, 

S.
Wright

, & 

M.
Johnston

 (Eds.), Measures in health psychology: A user's portfolio. Causal and control beliefs (pp. 35–37). NFER‐NELSON.

Snyder, C. R.
, 
Harris, C.
, 
Anderson, J. R.
, 
Holleran, S. A.
, 
Irving, L. M.
, 
Sigmon, S. T.
, 
Yoshinobu, L.
, 
Gibb, J.
, 
Langelle, C.
, & 
Harney, P.
 (1991). The will and the ways: Development and validation of an individual‐differences measure of hope. Journal of Personality and Social Psychology, 60, 570–585. 10.1037//0022-3514.60.4.570
2037968


Waterman, A. S.
 (2015). What does it mean to engage in identity exploration and to hold identity commitments? A methodological critique of multidimensional measures for the study of identity processes. Identity: An International Journal of Theory and Research, 15, 309–349. 10.1080/15283488.2015.1089403


Zhu, G.
, & 
Burrow, A. L.
 (2022). Youth voice in self‐driven learning as a context for interdisciplinary learning. Journal of Educational Studies and Multidisciplinary Approaches, 2, 132–154. 10.51383/jesma.2022.29


## Data Availability

The analyses presented here were not preregistered. Data, materials, and analytic code are available from the corresponding author upon reasonable request.
